# Sulfonyl-substituted zinc phthalocyanine micelles with long-lived triplet states enable durable photodynamic immunotherapy

**DOI:** 10.1039/d6na00602g

**Published:** 2026-08-11

**Authors:** Paweł Repetowski, Marta Warszyńska, Fabienne Dumoulin, Janusz M. Dąbrowski

**Affiliations:** a Faculty of Chemistry, Jagiellonian University Kraków 30-387 Poland jdabrows@chemia.uj.edu.pl; b Doctoral School of Exact and Natural Sciences, Jagiellonian University Kraków 30-348 Poland; c Faculty of Engineering and Natural Sciences, Department of Biomedical Engineering, Acıbadem Mehmet Ali Aydınlar University Ataşehir Istanbul 34752 Türkiye

## Abstract

Pluronic® P123 micelles stabilized a sulfonyl-substituted zinc(ii) phthalocyanine, prolonged its triplet-state lifetime, and preserved ROS photochemistry under biologically relevant conditions. Pharmacokinetic-guided C-PDT at a 24 h DLI remained insufficient as monotherapy, whereas combination with PD-L1 blockade produced durable tumor control. Rechallenge experiments after PDT additionally revealed complete long-term antitumor immune memory.

## Abstract 2

Pluronic® P123 micelles stabilized a sulfonyl-substituted zinc(ii) phthalocyanine, prolonged triplet lifetime, preserved ROS photochemistry, enabled PK-guided C-PDT, and supported durable tumor control with PD-L1 blockade. Created with BioRender.com.

## Graphical abstract

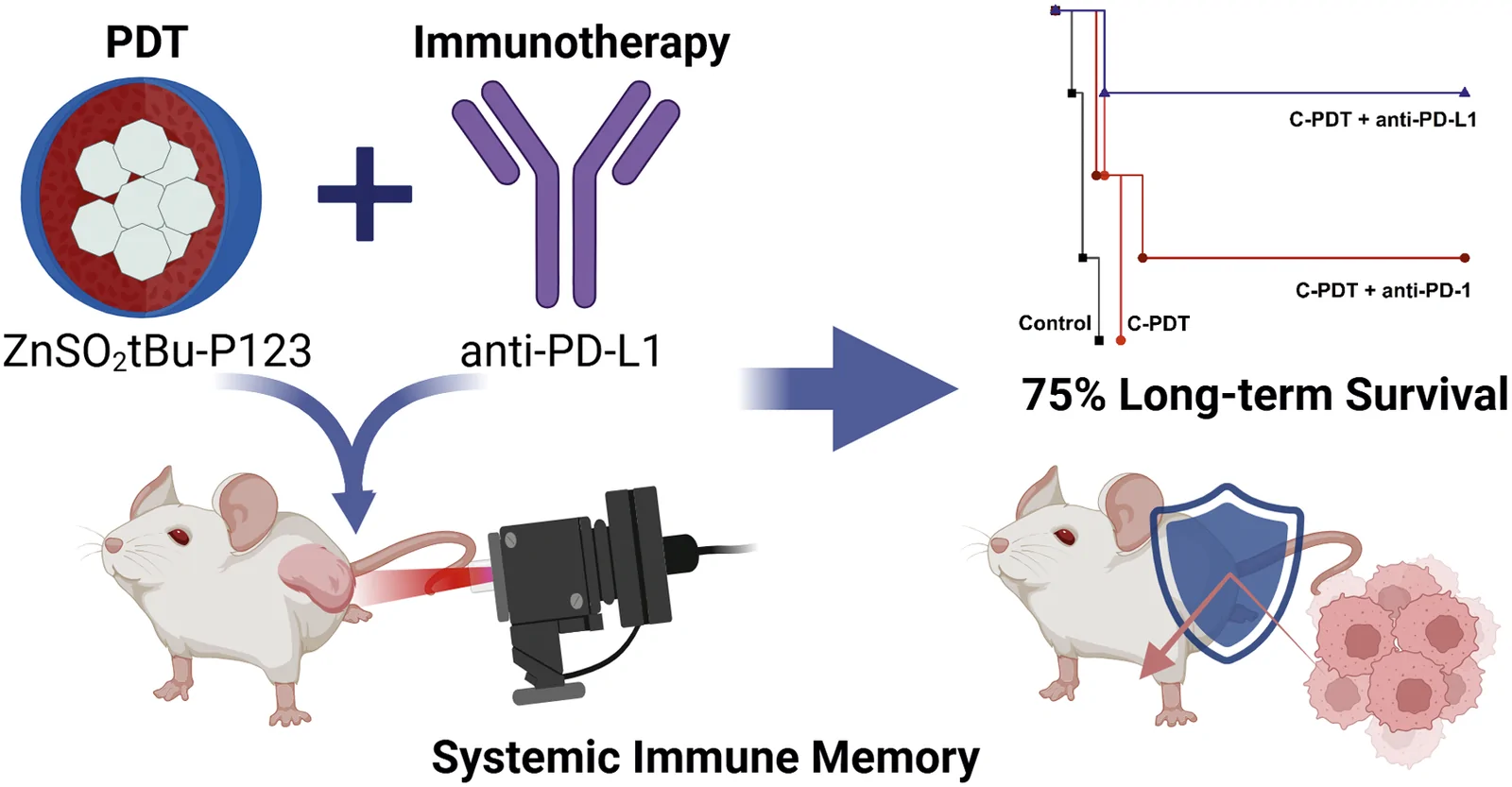

Photodynamic therapy (PDT) exploits the spatial coincidence of a photosensitizer, light, and dioxygen to generate cytotoxic reactive oxygen species (ROS); therefore, its therapeutic outcome depends not only on photochemistry, but also on formulation, biodistribution, drug-to-light interval (DLI), and the immune response.^1–4^ Phthalocyanines are among the most attractive PDT chromophores because of their strong red-light absorption and high photochemical performance;^5^ however, their biological translation for cellular-targeted photodynamic therapy (C-PDT) is frequently hindered by severe aqueous aggregation, poorly controlled pharmacokinetics, and limited immunological efficacy.^2,3,6^ Here, we address this limitation using a Pluronic® P123 micellar formulation of a sulfonyl-substituted zinc(ii) phthalocyanine (ZnSO_2_*t*Bu, Fig. S1A), which preserves the photoactive state of the macrocycle under biologically relevant conditions and enables pharmacokinetic-guided C-PDT combined with PD-L1 blockade.^7–11^ Sulfonyl-substituted metal phthalocyanines were previously introduced by our group as efficient ROS-generating photosensitizers for vascular-targeted PDT (V-PDT).^6^ In that study, both platinum(ii) (PtSO_2_*t*Bu, Fig. S1B) and zinc(ii) (ZnSO_2_*t*Bu) derivatives demonstrated high efficacy against CT26 tumors under short-DLI vascular protocols, with PtSO_2_*t*Bu producing complete local tumor eradication and ZnSO_2_*t*Bu achieving 84% cures. Although PtSO_2_*t*Bu led to stronger primary tumor control, new rechallenge experiments revealed a more pronounced systemic immune memory response for the ZnSO_2_*t*Bu. While our previous work described the chemistry and short-DLI V-PDT activity of sulfonyl-substituted metallophthalocyanines,^6^ the present study examines ZnSO_2_*t*Bu in the optimized Pluronic® P123 nanoformulation administered *in vivo*.

Using this formulation, we define fluorescence properties, ROS generation, and triplet-state dynamics in micelles, followed by serum pharmacokinetics, biodistribution, tumor-to-normal tissue ratios, and selection of a 24 h DLI for C-PDT. This longer-DLI protocol favours tumor accumulation while avoiding the immediate vascular shutdown typical of aggressive V-PDT, which may limit subsequent immune-cell access to the tumor.^12,13^ We therefore evaluated the immune-memory potential of ZnSO_2_*t*Bu-mediated PDT in a rechallenge experiment and tested whether pharmacokinetic-guided C-PDT could benefit from PD-1 or PD-L1 blockade. Classical liposomal formulations of unsubstituted ZnPc (Fig. S1C) established 24 h DLI conditions as favorable for maximizing tumor-to-normal tissue ratios in clinically relevant PDT protocols.^14,15^ However, despite substantial tumor accumulation, standalone C-PDT frequently remains insufficient to induce durable tumor eradication.^11^ We therefore investigated whether rational micellar stabilization of ZnSO_2_*t*Bu combined with PD-1/PD-L1 blockade could overcome this limitation. Self-assembly of ZnSO_2_*t*Bu with Pluronic® P123 (Fig. 1A) afforded a micellar formulation with a dominant nanoscale population, as shown by DLS (Z-average hydrodynamic diameter: 27.7 nm; PDI = 0.43, Fig. 1C). TEM supported the formation of spherical micellar nanostructures with occasional larger loose agglomerates (Fig. 1D and S2), likely from reversible Pluronic-associated aggregation and consistent with the secondary DLS intensity fraction. Volume- and number-weighted DLS distributions confirmed the predominance of the primary nanoscale population while resolving a minor larger-size fraction (Fig. S3). Accordingly, the formulation is described as a reproducible micellar system with nanoscale heterogeneity rather than as a monodisperse nanoparticle preparation. Only batches displaying reproducible DLS profiles were used for biological and *in vivo* experiments. The near-neutral *ζ*-potential of −2.51 ± 0.98 mV (Table 1) is consistent with a non-ionic poly(ethylene oxide) (PEO) corona and predominantly steric stabilization. Time-dependent stability of ZnSO_2_*t*Bu-P123 was evaluated in PBS and PBS containing 10% fetal bovine serum (FBS). In 10% FBS, DLS showed time-dependent size redistribution over 48 h, with the PDI decreasing from 0.49 to 0.19, indicating no progressive broadening or macroscopic aggregation. Absorption spectra showed moderate hypochromicity, retaining approximately 70% of the initial Q-band intensity after 48 h, while preserving a monomeric band profile without severe broadening or spectral shifts (Fig. S4). These data indicate resistance to rapid serum-induced aggregation and sufficient optical integrity over the 24 h DLI window, consistent with prolonged systemic exposure, preserved fluorescence, and progressive tumor accumulation *in vivo*. Encapsulation within the hydrophobic poly(propylene oxide) (PPO) core of P123 resolved the aqueous insolubility of ZnSO_2_*t*Bu, while the hydrophilic PEO shell provided steric stabilization in physiological media.^13^ The thin-film hydration method yielded an encapsulation efficiency of 88.0 ± 5.1%. Because unencapsulated ZnSO_2_*t*Bu is highly hydrophobic and essentially insoluble in water, it precipitates and is removed by 0.22 µm filtration; thus, the quantified photosensitizer predominantly corresponds to the micelle-associated fraction (Table 1 and Fig. S5). The photostability of the formulation was evaluated by monitoring Q-band absorbance decay under irradiation with a 652 nm laser matching the conditions used for *in vivo* studies (Fig. 1E).

**Fig. 1:**
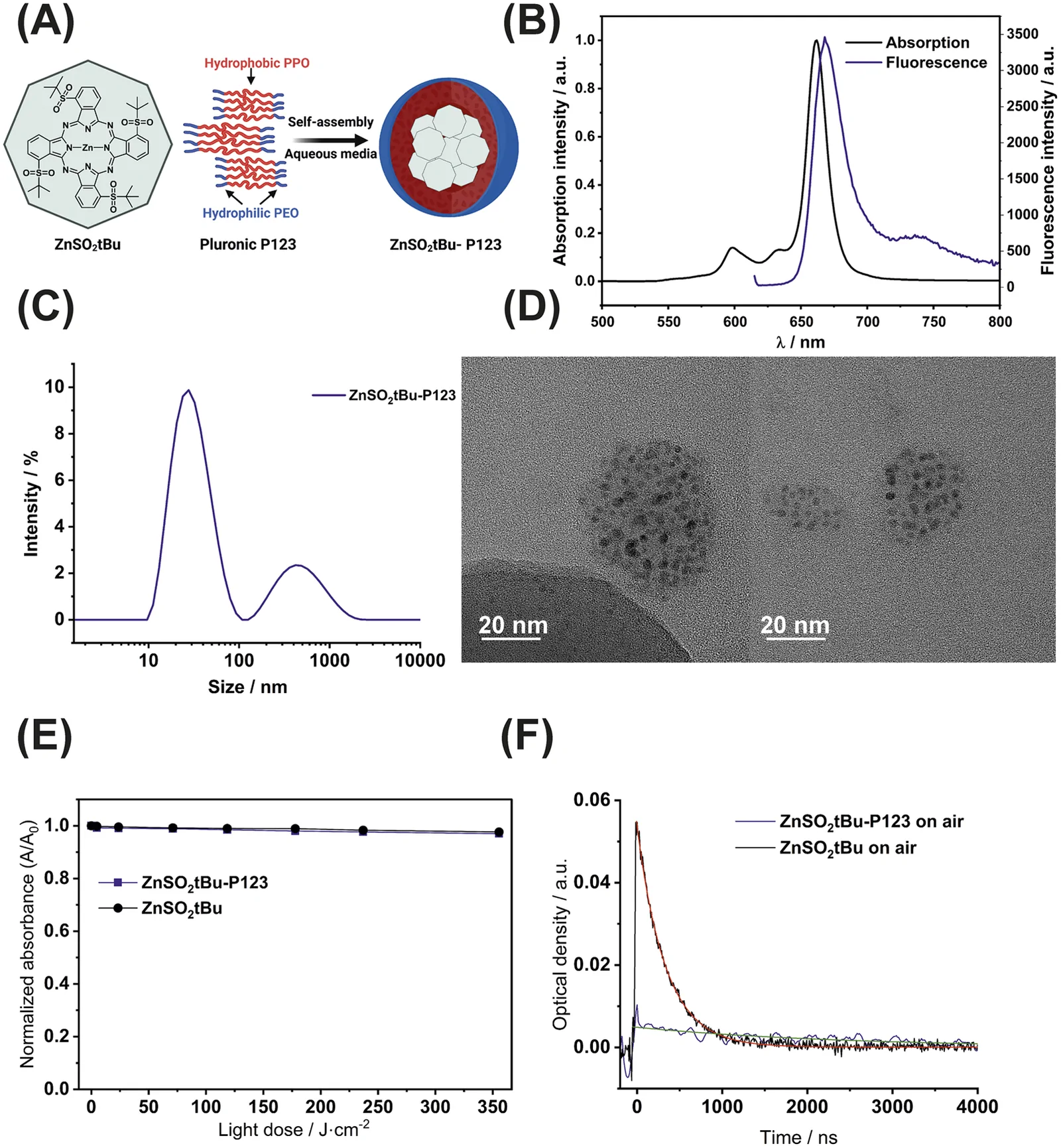
Preparation and physicochemical characterization of ZnSO_2_*t*Bu-P123 micelles. (A) Schematic illustration of micellar self-assembly. (B) UV-vis absorption and fluorescence spectra. (C) DLS size distribution profile (*Z*_ave_ = 27.7 nm). (D) TEM images supporting the formation of spherical micellar nanostructures. (E) Photostability of free ZnSO_2_*t*Bu and ZnSO_2_*t*Bu-P123 under 652 nm irradiation matching *in vivo* PDT conditions. (F) Transient absorption decay profiles under air-equilibrated conditions demonstrating prolonged triplet-state lifetime after micellar encapsulation.

**Table 1:** Key formulation, colloidal, photophysical, and photochemical parameters of ZnSO_2_*t*Bu and ZnSO_2_*t*Bu-P123 micelles^a^

	EE/%	ζ-potential/mV	*Φ*_F_	*τ*_F_/ns	*Φ*_Δ_	*τ*_T_/ns [air]
ZnSO_2_*t*Bu	—	—	0.172 ± 0.014 (ref. 6)	2.39 ± 0.01	0.45 ± 0.02 (ref. 6)	350
ZnSO_2_*t*Bu-P123	88.0 ± 5.1	−2.51 ± 0.98	0.127 ± 0.003	2.61 ± 0.01	0.54 ± 0.02	2300

a EE: encapsulation efficiency; *ζ*-potential: zeta potential; *Φ*_F_: fluorescence quantum yield; *τ*_F_: fluorescence lifetime; *Φ*_Δ_: singlet oxygen quantum yield; *τ*_T_ [air]: triplet-state lifetime under air-equilibrated conditions. Values marked with “(ref. 6)” were taken from ref. 6. Data are presented as mean ± SD where applicable.

ZnSO_2_*t*Bu-P123 showed higher resistance to photobleaching than the free dye, consistent with reduced aggregation and stabilization of the photoactive state within the micellar environment. In DMF, ZnSO_2_*t*Bu exhibited *Φ*_F_ = 0.172 ± 0.014 and *τ*_F_ = 2.39 ± 0.01 ns, whereas micellar ZnSO_2_*t*Bu-P123 in PBS showed lower *Φ*_F_ = 0.127 ± 0.003 and slightly longer *τ*_F_ = 2.61 ± 0.01 ns (Table 1 and Fig. S6). The lower *Φ*_F_ suggests a reduced contribution of radiative decay and a larger contribution of triplet/ROS-generating pathways, as supported by the ROS data below. The slightly longer *τ*_F_ is consistent with altered excited-state deactivation within the hydrophobic P123 core and reduced aggregation-induced quenching, while the retained emission enabled non-invasive *in vivo* tracking. The formulation also retained high photochemical activity in aqueous media. The singlet oxygen quantum yield (*Φ*_Δ_) determined using the Singlet Oxygen Sensor Green (SOSG) probe reached 0.54 ± 0.02 (Table 1 and Fig. S7A).^14^ Aminophenyl fluorescein (APF) oxidation indicates the formation of highly reactive ROS, supporting a contribution from Type I photochemistry (Fig. S7B).^15^ The combined SOSG and APF responses indicate that ZnSO_2_*t*Bu-P123 retains Type II photochemical competence while also supporting Type I-associated ROS formation under biologically relevant aqueous conditions. This dual-pathway behavior is important because Type II photosensitization is governed by triplet-state energy transfer to O_2_, whereas Type I processes involve electron- or hydrogen-transfer reactions that may contribute to oxidative damage when oxygen availability becomes restricted.^16^ Transient absorption spectroscopy confirmed triplet-state formation within the micellar formulation (Fig. S8). Under argon-saturated conditions, ZnSO_2_*t*Bu-P123 exhibited a triplet lifetime of approximately 40 µs, consistent with the long-lived excited-state behavior previously observed for ZnSO_2_*t*Bu in organic media.^6^ Under air-equilibrated conditions, *τ*_T_ reached 2.3 µs (Fig. 1F), approximately 6.6-fold longer than for the unformulated photosensitizer. For a highly hydrophobic phthalocyanine, micellar encapsulation provides a more biologically relevant nanoconfined environment than molecular dispersion in organic solvents, helping preserve triplet-state reactivity while limiting aggregation-driven deactivation. This microenvironment suppresses aggregation-driven and non-radiative deactivation pathways while maintaining sufficient oxygen accessibility for Type II energy transfer and parallel Type I-associated ROS generation.^17^

The air-equilibrated *τ*_T_ of ZnSO_2_*t*Bu-P123 compares favorably with reported values for established PDT photosensitizers, although direct comparison is limited by differences in formulation and measurement conditions.^18^ This supports the view that P123 preserves triplet-state reactivity in aqueous media, complementing its delivery function rather than independently predicting therapeutic efficacy. In contrast to the platinum analogue, the retained fluorescence of ZnSO_2_*t*Bu additionally enabled real-time non-invasive imaging and pharmacokinetic-guided optimization of the DLI.

The selection of ZnSO_2_*t*Bu for subsequent C-PDT studies was supported by a tumor rechallenge experiment performed 90 days after primary PDT (Fig. 2A). This recovery period minimized the contribution of local inflammation and enabled evaluation of long-term adaptive immunity. To assess whether the immune response was systemic, cured mice were reinoculated with CT26 cells on the contralateral limb. Remarkably, the ZnSO_2_*t*Bu-P123 treated group showed complete tumor rejection in all animals (Fig. 2B), demonstrating potent stimulation of long-term antitumor immune memory. These observations are consistent with immunogenic mechanisms associated with intracellular oxidative stress.^19^ Similar effects have been reported for other photosensitizers, where intracellular ROS generation promoted ER stress and DAMP release.^20–23^ The ability of ZnSO_2_*t*Bu-P123 to induce durable immune protection provided the rationale for focusing the subsequent studies on a cellular-targeted PDT regimen combined with immune checkpoint blockade. Transitioning from vascular to cellular PDT required analysis of the systemic fate and biodistribution of ZnSO_2_*t*Bu-P123*in vivo*. Blood pharmacokinetic (PK) data were analyzed using Certara Phoenix WinNonlin software supported by goodness-of-fit diagnostic plots (Fig. S9). The concentration–time profile exhibited biphasic decay kinetics (Fig. 3A), consisting of an initial distribution phase followed by slower elimination, and was adequately described using non-compartmental analysis (NCA) and a one-compartment model. The micellar formulation displayed an elimination half-life (*t*_1/2_) of 14.86 ± 2.05 h, clearance of 0.057 L h^−1^ kg^−1^, and AUC_0-last_ of 18.92 ± 4.18 mg h^−1^ L^−1^ (Table 2).

**Fig. 2:**
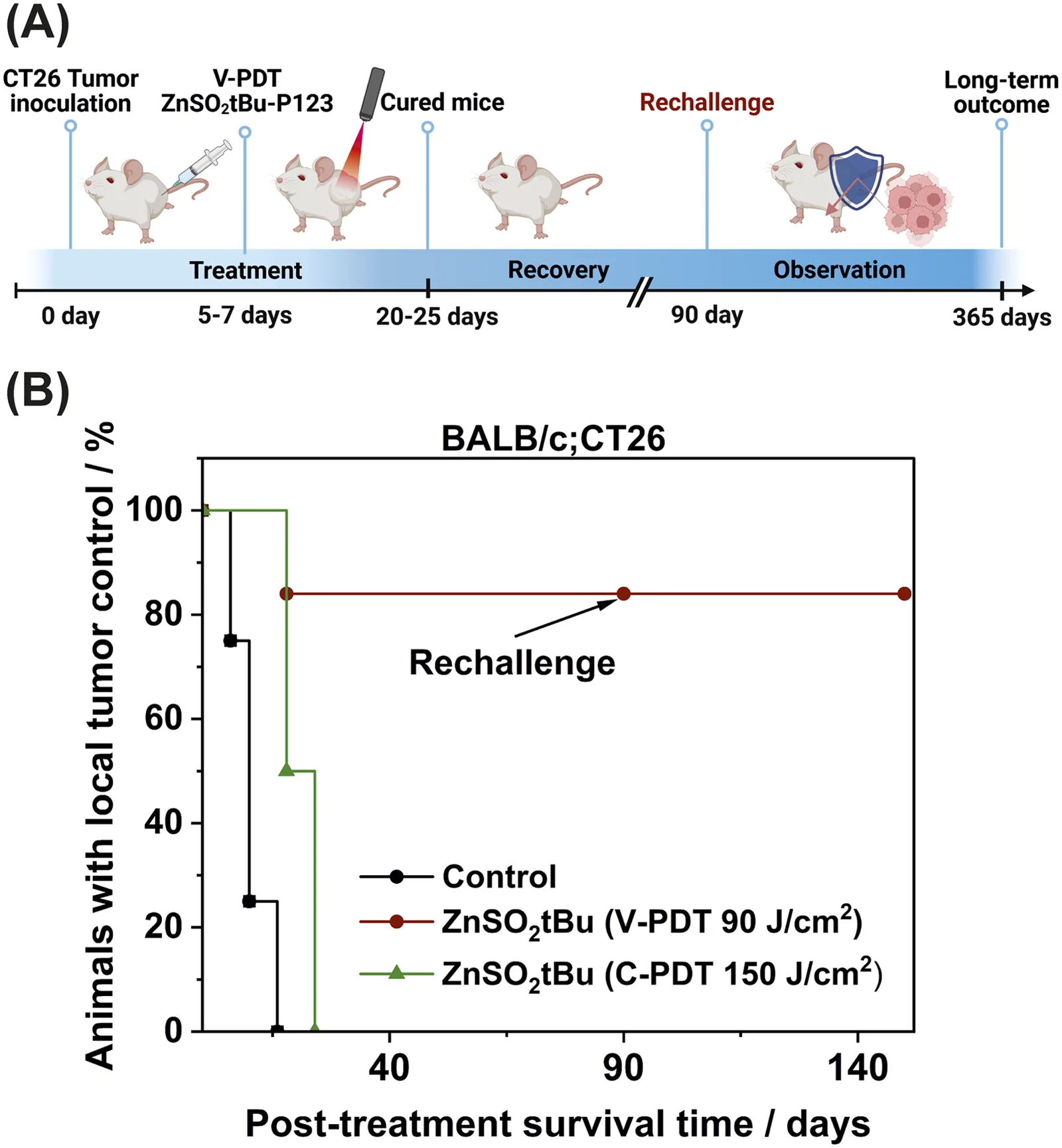
Long-term antitumor immune memory induced by ZnSO_2_*t*Bu-mediated photodynamic therapy. (A) Experimental design of the tumor rechallenge study. Mice cured after primary PDT were reinoculated with CT26 cells on the contralateral flank 90 days after treatment. Created with BioRender.com. (B) Kaplan–Meier analysis following rechallenge, demonstrating complete tumor rejection in mice previously treated with ZnSO_2_*t*Bu-mediated PDT (*p* = 0.0002, log-rank (Mantel–Cox) test).

**Fig. 3:**
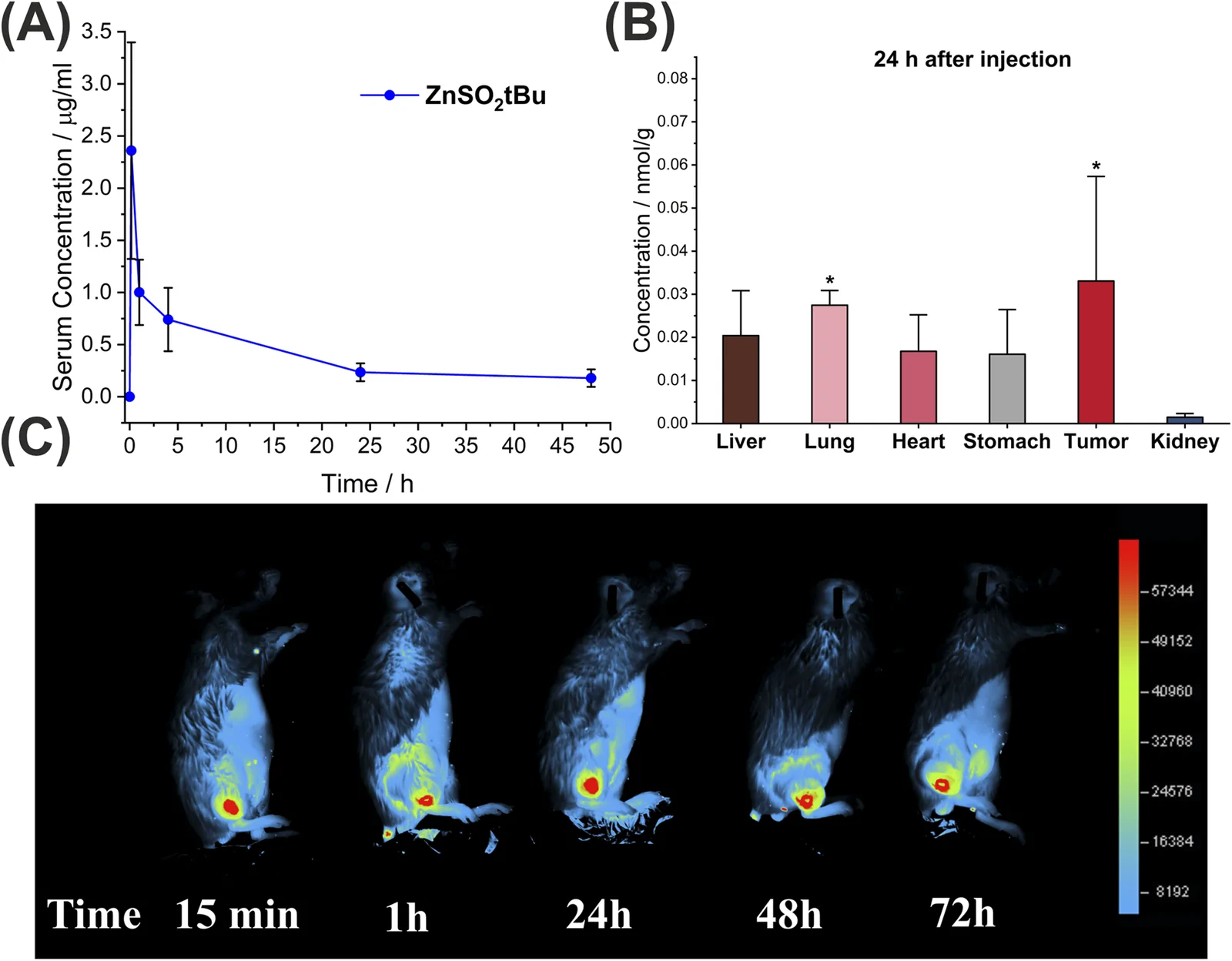
Pharmacokinetic, biodistribution, and *in vivo* imaging profiles of ZnSO_2_*t*Bu-P123. (A) Serum concentration – time profile (mean ± SEM) showing biphasic decay kinetics. (B) Quantitative *ex vivo* biodistribution at 24 h post-injection (mean ± SEM, *n* = 3). Tumor (*p* = 0.0121) and lung (*p* = 0.0335) accumulation exceeded untreated tissue autofluorescence. (C) Whole-body fluorescence imaging of CT26 tumor-bearing mice over 72 h visualizing the spatiotemporal distribution of ZnSO_2_*t*Bu-P123.

Pharmacokinetic parameters of the ZnSO_2_*t*Bu-P123 micellar formulation following intravenous administration in mice, calculated using non-compartmental analysis (NCA) and a 1-compartment model. Data are presented as mean ± SD

**Table:** 

Non-compartmental analysis (NCA)	
Parameter	Estimate
*C*_max_ (mg L^−1^)	2.36 ± 1.04
*t*_max_ (min)	10
AUC_0-last_ (mg h^−1^ L^−1^)	18.92 ± 4.18
AUC_0-∞_ (mg h^−1^ L^−1^)	23.78
MRT (h)	13.89

**Table:** 

1-Compartment model			
Parameter	Estimate	CV%	95% CI
*V*_D_ (L kg^−1^)	1.24 ± 0.31	24.7	0.58–1.89
Cl (L h^−1^ kg^−1^)	0.057 ± 0.013	23.0	0.029–0.086
*t*_1/2_ (h)	14.86 ± 2.05	13.8	10.46–19.26

This sustained systemic exposure supports good *in vivo* performance of the P123 micellar formulation.

Time-dependent biodistribution studies based on tissue homogenates (Fig. S10) and *ex vivo* whole-organ fluorescence imaging (Fig. S11) demonstrated progressive tumor accumulation following systemic administration. During the initial post-injection phase, the highest signal was detected in the liver and lungs, consistent with partial uptake by the reticuloendothelial system. Over time, the distribution gradually shifted toward the tumor. Tumor-to-normal tissue ratio (TNR) analysis (Fig. S12) confirmed progressive improvement in tumor selectivity over time, with clearly favorable tumor-to-organ ratios already established at 24 h post-injection. This time point was therefore selected as a clinically relevant C-PDT interval rather than a vascular-targeted protocol. At this time point (Fig. 3B), the tumor displayed the highest absolute accumulation of ZnSO_2_*t*Bu (∼0.033 nmol g^−1^), which significantly exceeded the untreated tissue autofluorescence (*p* = 0.0121). A significant signal above background was also detected in the lungs (∼0.028 nmol g^−1^, *p* = 0.0335). Although the mean accumulation in the tumor was higher than in other organs like the liver (∼0.020 nmol g^−1^), direct inter-organ differences lacked statistical significance due to the expected biological variance in the *n* = 3 cohort. Negligible accumulation within the kidneys indicates that ZnSO_2_*t*Bu-P123 is not efficiently cleared through glomerular filtration. Instead, the pharmacokinetic profile together with the initial hepatic uptake suggests predominant hepatobiliary processing of the formulation.^24,25^ The spatiotemporal distribution was further visualized by real-time whole-body fluorescence imaging (Fig. 3C), confirming progressive tumor accumulation over time. Guided by these biodistribution dynamics and the established use of 24 h intervals in cellular PDT, this DLI was selected for the C-PDT regimen. Notably, our previous PDT studies demonstrated that maximal tumor selectivity at longer DLIs does not necessarily translate into a superior therapeutic outcome, likely reflecting the interplay between pharmacokinetics and antitumor immunity during PDT.^26^ Despite favorable tumor accumulation, isolated C-PDT was insufficient to produce durable tumor eradication, resulting in relapse and 100% mortality by day 24 post-irradiation (Fig. 4B). Thus, C-PDT alone was insufficient to sustain long-term anticancer control despite favorable tumor accumulation. To overcome tumor-associated immunosuppression, C-PDT was combined with immune checkpoint inhibitors targeting PD-1 and PD-L1 (Fig. 4A). Antibodies were administered in a multidose regimen initiated on the day of irradiation and continued every three days. Combination therapy markedly improved the therapeutic outcome, with C-PDT combined with PD-L1 blockade producing the most favorable long-term survival (75%) compared with the C-PDT + anti-PD-1 regimen (25%). This difference is consistent with adaptive immunoregulatory mechanisms triggered by PDT-induced inflammatory signalling within the tumor microenvironment (TME),^27^ although the precise molecular determinants require further investigation. PDT-induced inflammatory signalling may promote adaptive PD-L1 expression in surviving cancer cells and tumor-associated macrophages, a resistance mechanism reported in PDT/immunotherapy settings.^28,29^ Anti-PD-L1 treatment may therefore limit PD-1/PD-L1-mediated suppression of recruited cytotoxic T cells. As PD-L1 dynamics, immune-cell infiltration, ICD markers, and DAMP release were not directly profiled here, this mechanism remains under investigation. Thus, the improved survival after C-PDT plus PD-L1 blockade is interpreted as therapeutic cooperation rather than direct proof of a defined immune pathway.

**Fig. 4:**
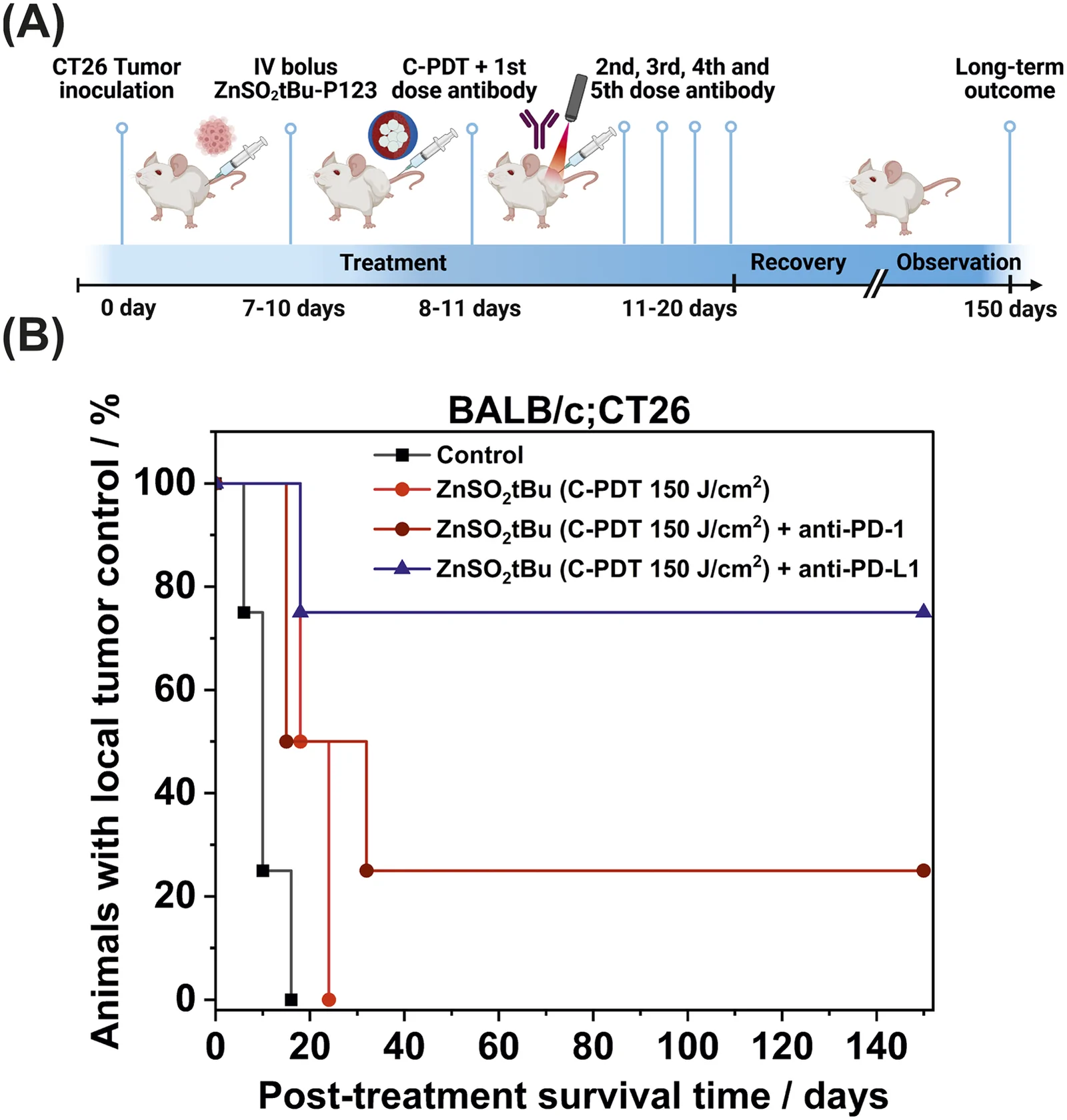
Therapeutic efficacy of cellular-targeted PDT combined with immune checkpoint blockade in CT26 tumors. (A) Treatment schedule showing ZnSO_2_*t*Bu-P123 administration, laser irradiation (150 J cm^−2^), and multidose antibody treatment. Created with BioRender.com. (B) Kaplan–Meier survival curves demonstrating superior long-term survival for the anti-PD-L1 combination therapy compared with anti-PD-1 treatment and standalone C-PDT (overall *p* < 0.0001, log-rank (Mantel–Cox) test).

This work identifies the Pluronic® P123 micellar delivery of ZnSO_2_*t*Bu as a formulation-based strategy linking aggregation control, triplet-state preservation, pharmacokinetic-guided PDT, and PD-L1 blockade. Under a PK-guided 24 h DLI, ZnSO_2_*t*Bu-P123 enabled C-PDT but remained insufficient as monotherapy, indicating that local treatment alone did not sustain long-term tumor control in this model. Rechallenge experiments demonstrated complete rejection of secondary CT26 tumors in all previously cured animals, highlighting the immune-memory potential of ZnSO_2_*t*Bu-mediated PDT. Combining C-PDT with PD-L1 blockade improved this otherwise transient response and produced durable tumor control, providing proof of concept for phthalocyanine-based photodynamic immunotherapy. Together, these findings show that a reproducibly prepared Pluronic® P123 micellar formulation, characterized and used consistently across the study, can preserve the photoactive state of ZnSO_2_*t*Bu and support PK-guided tumor delivery sufficient for long-term therapeutic benefits. Detailed studies of ICD markers, immune-cell infiltration, DAMP release, and PD-L1 dynamics are underway to define the immunological basis of this therapeutic cooperation.

## Ethical statement

All *in vivo* experiments were performed in accordance with ethical guidelines and were approved by the 2nd Local Institutional Animal Care and Use Committee (IACUC) in Kraków, Poland (Approval No. 242/2022).

## Conflicts of interest

There are no conflicts to declare.

## Supplementary Material

NA-008-D6NA00602G-s001

## Data Availability

All data supporting the findings of this study are available within the article and its supplementary information (SI). Supplementary information: experimental procedures, additional DLS/TEM characterization, stability, fluorescence lifetime, ROS generation, triplet-state decay, pharmacokinetic diagnostics, bio distribution, *ex vivo* imaging, and TNR analyses. See DOI: https://doi.org/10.1039/d6na00602g.
