# Supplementary material for: Sulfonyl-substituted zinc phthalocyanine micelles with long-lived triplet states enable durable photodynamic immunotherapy
Source: Nanoscale Adv. 2026 Aug 11;8(18):5102–7. doi: 10.1039/d6na00602g (PMC13483066; doi:10.1039/d6na00602g)
Supplement: Supplementary file 1 [file NA-008-D6NA00602G-s001.pdf]

Supplementary Information (SI) for Nanoscale Advances.
This journal is © The Royal Society of Chemistry 2026

Supporting Information

Sulfonyl-substituted zinc phthalocyanine micelles with long-lived triplet states enable durable photodynamic immunotherapy

Paweł Repetowski,[a,b] Marta Warszyńska,[a,b] Fabienne Dumoulin,[c] and Janusz M. Dąbrowski[*,a]

[a] *Faculty of Chemistry, Jagiellonian University, 30-387 Kraków, Poland.*

[b] *Doctoral School of Exact and Natural Sciences, Jagiellonian University, 30-348 Kraków, Poland.*

[c] *Faculty of Engineering and Natural Sciences, Department of Biomedical Engineering, Acıbadem Mehmet Ali Aydınlar University, Ataşehir, Istanbul 34752, Türkiye*

Table of contents

Experimental procedures......S2

Fig. S1 Chemical structures of the **$ZnSO_2tBu$, $PtSO_2tBu$** and **ZnPc**......S7

Fig. S2 Wide-field transmission electron microscopy (TEM) image of **$ZnSO_2tBu$-P123**......S8

Fig. S3 Nanoscale micellar population dominates......S9

Fig. S4 Time-dependent colloidal and optical stability S9

Fig. S5 Linear calibration curve for the quantification of Encapsulation Efficiency (EE)......S10

Fig. S6 Time-resolved fluorescence decay profile......S10

Fig. S7 ROS generation kinetics utilizing SOSG and APF probes......S11

Fig. S8 Triplet state decay profiles......S11

Fig. S9 Goodness-of-fit diagnostic plots for pharmacokinetic modeling in mouse serum......S12

Fig. S10 Quantitative time-course *ex vivo* tissue biodistribution......S12

Fig. S11 *Ex vivo* fluorescence imaging of harvested organs......S13

Fig. S12 Tumor-to-normal tissue concentration ratios (TNR) over time......S13

Experimental Procedures

Preparation of photosensitizer-loaded polymeric micelles

The micellar formulation was prepared using the thin-film hydration method. Briefly, Pluronic P123 block copolymer was dissolved in ethanol at 50 °C, while the photosensitizer (**$ZnSO_2tBu$**) was dissolved in tetrahydrofuran (THF). Both solutions were then combined in a round-bottom flask and thoroughly mixed. The organic solvents were subsequently evaporated under reduced pressure using a rotary evaporator, resulting in the formation of a homogeneous, dry polymer-drug film on the flask wall. The film was further dried under vacuum overnight to ensure complete solvent removal. The thin film was then rehydrated with 5 mL of sterile phosphate-buffered saline (PBS, pH 7.4), followed by gentle sonication for 15 minutes to facilitate the self-assembly of the micellar formulation. To remove any unencapsulated photosensitizer aggregates and simultaneously ensure the sterility of the formulation, the resulting micellar solution was passed through a sterile 0.22 μm syringe filter. The purified micellar suspension was stored in the dark at 4 °C until further use. To ensure rigorous batch-to-batch reproducibility, the hydrodynamic diameters of the micellar suspensions were routinely validated via DLS prior to every *in vivo* administration. Only formulations displaying reproducible DLS profiles were used for the animal experiments, ensuring consistent dosing and comparable pharmacokinetic behavior.

Size distribution and surface charge analysis

The hydrodynamic dimensions and ζ-potential of the photosensitizer-encapsulated P123 nanocarriers were evaluated via dynamic light scattering (DLS) employing a Malvern Zetasizer instrument.

Determination of Encapsulation Efficiency (EE)

The encapsulation efficiency of **$ZnSO_2tBu$** within the Pluronic P123 micelles was quantified using UV-Vis spectrophotometry. Following the self-assembly process, the micellar suspension was filtered through a sterile 0.22 μm syringe filter to eliminate any unencapsulated photosensitizer aggregates. Subsequently, a defined aliquot of the purified micellar solution was diluted 20-fold in pure dimethyl sulfoxide (DMSO) and subjected to ultrasonication for 2 minutes. This procedure completely disrupted the polymeric core, releasing the macrocycle into its photoactive, monomeric state. The absorbance of the resulting solution was measured at the Q-band maximum. The precise concentration of the encapsulated dye was determined utilizing a predefined linear calibration curve (2 - 20 μg/mL) established for the **$ZnSO_2tBu$** in DMSO. The EE% was calculated as the mass ratio of the experimentally quantified photosensitizer in the filtered micelles to the initial amount of the drug used for the formulation.

Photochemical and Physiological Stability Assays

The structural and photochemical robustness of the formulation was evaluated under both continuous laser irradiation and prolonged dark incubation. To assess photobleaching resistance, solutions of **$ZnSO_2tBu$-P123** (in PBS) and free **$ZnSO_2tBu$** (in PBS containing 10% DMSO) were optically matched to an initial Q-band absorbance of approximately 0.88. Samples were irradiated in standard quartz cuvettes using a 652 nm diode laser, replicating the in vivo light

parameters, with stability expressed as the normalized absorbance (A/A_0) at the Q-band maximum against total irradiation time. Furthermore, to evaluate time-dependent colloidal and optical stability, micellar suspensions were incubated in the dark at room temperature for up to 48 h in PBS supplemented with 10% fetal bovine serum (FBS). UV-Vis absorption spectra were recorded at designated intervals to monitor Q-band intensity and spectral shape, while DLS measurements in 10% FBS were performed to follow serum-induced changes in hydrodynamic size distribution and PDI.

Hydroxyl Radical (•OH) Generation Assay (APF)

The aminophenyl fluorescein (APF) probe was utilized for the probe-based detection of highly reactive oxygen species. The observed fluorescence increment primarily reflects a hydroxyl radical-associated signal, serving as an indicator of Type I photochemistry, rather than an absolute mechanistic proof. To ensure a rigorous comparison, the **$ZnSO_2$tBu-P123** micelles (in pure PBS) and the free unformulated **$ZnSO_2$tBu** (in PBS supplemented with 10% DMSO to prevent immediate macroscopic precipitation of the highly hydrophobic macrocycle) were prepared at equimolar photosensitizer concentrations (17.6 μg/mL). The APF reagent was added to the solutions, which were subsequently transferred to a black 96-well microplate. The samples were irradiated with a 652 nm laser, and the fluorescence intensity of the APF probe (λ_{ex} = 490 nm, λ_{em} = 515 nm) was recorded using a TECAN microplate reader. To account for differences in the optical cross-section between the monodispersed micelles and the partially aggregated free dye, the relative APF fluorescence increment ($F/F_0 - 1$) was mathematically normalized to the fraction of light effectively absorbed ($1 - 10^{-A}$) by each specific sample at the irradiation wavelength.

Singlet Oxygen Quantum Yield (Φ_Δ) Determination

The singlet oxygen generation was quantified utilizing the Singlet Oxygen Sensor Green (SOSG) standard. The measurements were conducted using a comparative method relative to a well-characterized reference photosensitizer (Methylene blue, Φ_Δ = 0.52 in water). Solutions of **$ZnSO_2$tBu-P123** and the reference dye were optically matched at the excitation wavelength. Upon the addition of the SOSG probe, the samples were irradiated, and the increase in SOSG fluorescence emission (λ_{ex} = 504 nm, λ_{em} = 525 nm) was monitored as a function of the delivered light dose. The quantum yield (Φ_Δ) calculations were strictly corrected for the absorbance of the micellar formulation at the excitation wavelength to prevent inner-filter effects, comparing the linear slope of the normalized fluorescence growth.

Triplet state lifetime measurements

Laser flash photolysis was employed to evaluate the triplet state lifetimes. Measurements were conducted at 20 °C in aqueous solutions. Deoxygenation was achieved by purging the samples with argon for 20 minutes prior to measurement. Decay kinetics were monitored at 480 nm following excitation at 355 nm. Data represent the average of 10 independent laser shots. The triplet lifetimes were determined by applying a monoexponential fit to the baseline-corrected decay curves.

Fluorescent lifetime

Time-resolved fluorescence decays were acquired via the Time-Correlated Single Photon Counting (TCSPC) technique employing a Fluorolog-3 spectrofluorometer (Horiba Jobin-Yvon). Excitation was provided by a 605 nm pulsed laser diode. The emission decays were monitored at the respective fluorescence maxima for each sample, with data collection continuing until 10,000 counts were accumulated in the peak channel. The instrument response function (IRF) was determined using a dilute aqueous suspension of colloidal silica (LUDOX) as a scattering medium. Decay profiles for both the free **$ZnSO_2tBu$** in DMF and the micellar **$ZnSO_2tBu$-P123** formulation in PBS were processed using DAS6 software (version 6.4, Horiba Jobin-Yvon). The experimental data were successfully fitted to a bi-exponential decay model, yielding reduced chi-square (χ^2) values in the range of 1.4–1.6, which was corroborated by a random, symmetrical distribution of residuals around the zero baseline.

Transmission Electron Microscopy (TEM) imaging

The morphological characteristics and size of the formulated **$ZnSO_2tBu$-P123** were evaluated using Transmission Electron Microscopy (TEM). Briefly, a small drop of the diluted micellar suspension was deposited onto a carbon-coated copper grid. The excess liquid was carefully blotted with filter paper, and the sample was allowed to air-dry completely at room temperature. The grids were subsequently examined, and images were captured utilizing a FEI Tecnai Osiris transmission electron microscope operating at an accelerating voltage of 200 kV.

Animal model and ethical statement

All *in vivo* experiments were performed in accordance with ethical guidelines and were approved by the 2nd Local Institutional Animal Care and Use Committee (IACUC) in Kraków, Poland (Approval No. 242/2022). Male BALB/c inbred mice (10 - 12 weeks old) were purchased from a certified breeder (AnimaLab Sp. z o.o., Poland). The animals were treated humanely and provided with standard pelleted feed and drinking water *ad libitum*. The mice were housed in individually ventilated cages (IVC) under Specific Pathogen Free (SPF) conditions at the animal facility of the Faculty of Biochemistry, Biophysics and Biotechnology, Jagiellonian University in Kraków. Strictly controlled environmental conditions were maintained: temperature at 22 ± 2 °C, relative humidity of 50 - 60%, and a standard 12 h light/12 h dark cycle.

The mean tumor diameter (θ) was calculated based on three mutually perpendicular dimensions using the following equation:

$$\theta = \sqrt[3]{a \cdot b \cdot c}$$

where a, b, c represent the measured tumor dimensions. For all systemic treatments, the micellar formulations (P123) were administered as a single intravenous (IV) bolus injection via the lateral tail vein in a constant volume of 100 μL.

In vivo and ex vivo fluorescence imaging

The biodistribution of the micellar systems was evaluated using whole-body fluorescence imaging. Once the subcutaneously implanted tumors reached approximately 0.5 cm in diameter, the animals received an intravenous (IV) bolus of the **$ZnSO_2tBu$-P123** at a dose of 1.5 mg/kg body weight. Imaging was performed using the Newton 7.0 small animal imaging system (Vilber, France). To isolate the specific fluorescence signal of the phthalocyanines, the optical

parameters were set to an excitation wavelength (λ_{ex}) of 680 nm and an emission range (λ_{em}) of 740–780 nm.

Real-time *in vivo* imaging was conducted at multiple designated time points (immediately post-injection, 1 h, 4 h, 24 h, 48 h, and 72 h) to monitor whole-body fluorescence kinetics. During these imaging procedures, mice were kept under general anesthesia via an intraperitoneal (IP) injection of a ketamine (75 mg/kg, Bioketan, Vetoquinol Biowet) and medetomidine (1 mg/kg, Domitor, Orion Pharma) mixture. Following the imaging sessions, anesthesia was pharmacologically reversed using IP atipamezole (Antisedan, Orion Corporation).

For terminal ex vivo organ imaging and quantitative biodistribution, animals were euthanized at designated time points up to 48 h post-injection. *Ex vivo* imaging of the harvested organs (heart, lungs, liver, kidneys, stomach, and tumor) was immediately performed to provide a direct visual comparison of the drug payload across different tissues. Background autofluorescence was subtracted using pre-injection images to ensure signal specificity.

In vivo Photodynamic Therapy (PDT), Rechallenge and Immunotherapy

To establish the solid tumor model, CT26 murine colon carcinoma cells ($0.35 \cdot 10^6$ cells suspended in 100 µL of PBS) were inoculated subcutaneously into the right thigh of BALB/c mice. Upon reaching a tumor diameter of 4 - 5 mm (7 - 10 days post-inoculation), the animals were randomly assigned to experimental groups. The standalone PDT cohort consisted of n = 6 mice, whereas the combination therapy cohorts receiving immune checkpoint inhibitors consisted of n = 4 mice per group.

The mice received an IV administration of the **$ZnSO_2$tBu-P123**. Tumor irradiation was performed utilizing two distinct drug-to-light intervals (DLI): 15 minutes (for V-PDT) and 24 hours (for C-PDT). An Omicron diode laser (PDT652.2-500) emitting at 652 nm was used as the light source, with a constant spot size of 1.3 cm in diameter and an irradiance of 80 mW/cm^2. The total light dose was 90 J/cm^2 for the 15-minute DLI scheme and 150 J/cm^2 for the 24-hour DLI scheme.

To evaluate the development of long-term systemic immune memory, mice that were completely cured following the primary vascular-targeted photodynamic therapy (V-PDT) were subjected to a tumor rechallenge. On day 90 post-treatment, the cured mice (n = 5) and age-matched naive control mice (n = 5) were subcutaneously re-inoculated with $0.35 \cdot 10^6$ CT26 murine colon carcinoma cells suspended in 100 µL of PBS into the contralateral flank. Tumor growth and survival were continuously monitored using the identical parameters established for the primary tumor model.

For the combination therapy (C-PDT + immunotherapy) groups, mice were treated with either anti-PD-1 (clone RMP1-14) or anti-PD-L1 (clone 7-H1) monoclonal antibodies, both sourced from Bio X Cell. The antibodies were administered via intraperitoneal (IP) injection at a dose of 2.5 mg/kg body weight. Treatment was initiated on the day of PDT irradiation (Day 0) and repeated every 3 days for a total of five doses.

Post-treatment monitoring included regular body weight measurements, tumor growth dynamics assessment, and behavioral observations. Animals were humanely euthanized when tumors exceeded 10 mm in diameter. Therapeutic efficacy was evaluated using Kaplan-Meier survival curves.

Biodistribution and Pharmacokinetic Sampling

A sparse terminal sampling design was employed to evaluate both the systemic pharmacokinetic (PK) profile and the organ biodistribution. Time points were set at 10 min, 1 h, 4 h, 24 h, and 48 h post-administration. At each designated time point, animals were humanely euthanized. Blood was collected via cardiac puncture, allowed to clot at room temperature for 30 - 45 minutes, and centrifuged (12000 x g, 15 min) to obtain serum. Simultaneously, major organs (heart, lungs, liver, stomach, kidneys) and the tumor tissue were harvested, rinsed with ice-cold PBS, weighed, and immediately frozen at -80 °C for further analysis.

Tissue Processing and Drug Extraction

To quantify the phthalocyanine content in the collected tissues, the organs were subjected to mechanical disruption. Each tissue sample was homogenized in a specific volume of PBS buffer using a Yellowline IKA DI 25 disperser to obtain uniform tissue homogenates.

To release the **$ZnSO_2tBu$** from the micellar structures and biological matrices, a solvent-based extraction (protein precipitation) was performed for both serum and tissue homogenates. An extraction mixture consisting of ethanol and dimethyl sulfoxide (DMSO) (75:25, v/v) was added to the samples at a 1:9 ratio (e.g., 50 µL of serum or homogenate to 450 µL of extraction mixture). The samples were vortexed vigorously for 1 minute, sonicated in an ultrasonic bath for 10 minutes, and centrifuged (14,000 x g, 15 min). The clear supernatant containing the extracted photosensitizer was isolated for quantitative analysis.

Fluorescence Quantification and Analysis

The total concentration of **$ZnSO_2tBu$** in the supernatants was measured using black 96-well microplates and a TECAN Infinite 200 Pro microplate reader (Tecan, Switzerland). The fluorescence intensity was recorded at an excitation wavelength (λ_{ex}) of 600 nm and an emission wavelength (λ_{em}) of 667 nm.

Standard curves (range 0 - 50 µg/mL) were prepared separately for serum and for each tissue type by spiking blank biological matrices with known concentrations of the free phthalocyanine to account for matrix-specific quenching effects.

Pharmacokinetic parameters were estimated using Phoenix WinNonlin (version 8.5.2.4, Certara, USA) via the Naive Pooled Data approach. For biodistribution, the drug concentration in each organ was expressed as nmol/g of tissue.

Statistical analysis

Data are presented as mean ± SD or SEM, as indicated in the figure legends. For the quantitative ex vivo biodistribution (Fig. 3B), the accumulation in treated organs was compared against the autofluorescence of untreated controls using an ordinary two-way ANOVA (without matching or pairing), followed by the uncorrected Fisher's LSD test. Time-course biodistribution across multiple organs (Fig. S10) was evaluated using two-way ANOVA with Dunnett's multiple comparisons test. Kaplan-Meier survival curves (Fig. 2B and Fig. 4B) were compared using the log-rank (Mantel-Cox) test. Pharmacokinetic modeling was not subjected to inferential group

comparison because a single formulation was evaluated; goodness-of-fit diagnostics and model-derived parameters are provided in Fig. S9 and Table 2. $P < 0.05$ was considered statistically significant. Exact P values are reported in the text or corresponding figure legends. Statistical analyses were performed using GraphPad Prism (8.0.1).

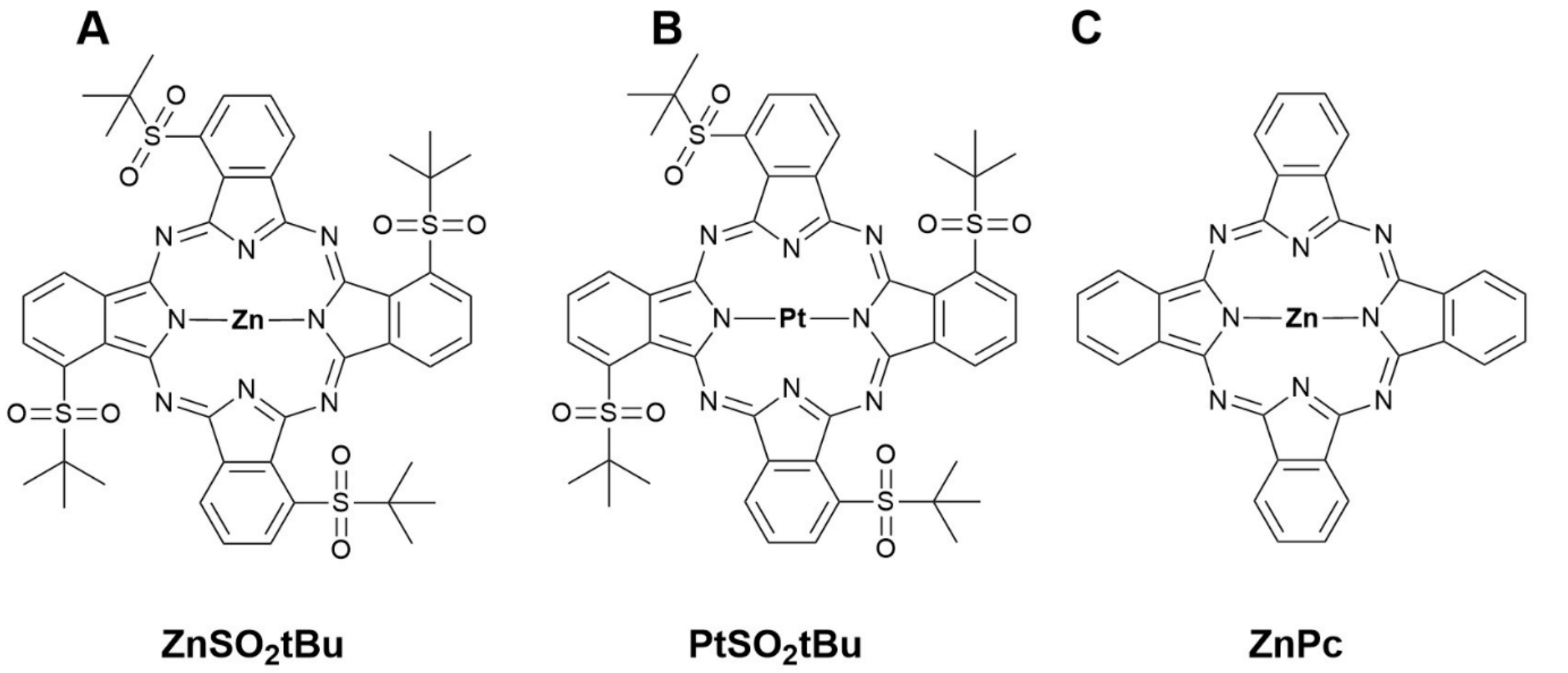

Fig. S1. Chemical structures of the photosensitizers discussed in this work: (A) the investigated sulfonyl-substituted zinc(II) phthalocyanine (**$ZnSO_2tBu$**), (B), the sulfonyl-substituted platinum(II) phthalocyanine (**$PtSO_2tBu$**) and (C) classical unsubstituted zinc(II) phthalocyanine (**ZnPc**).

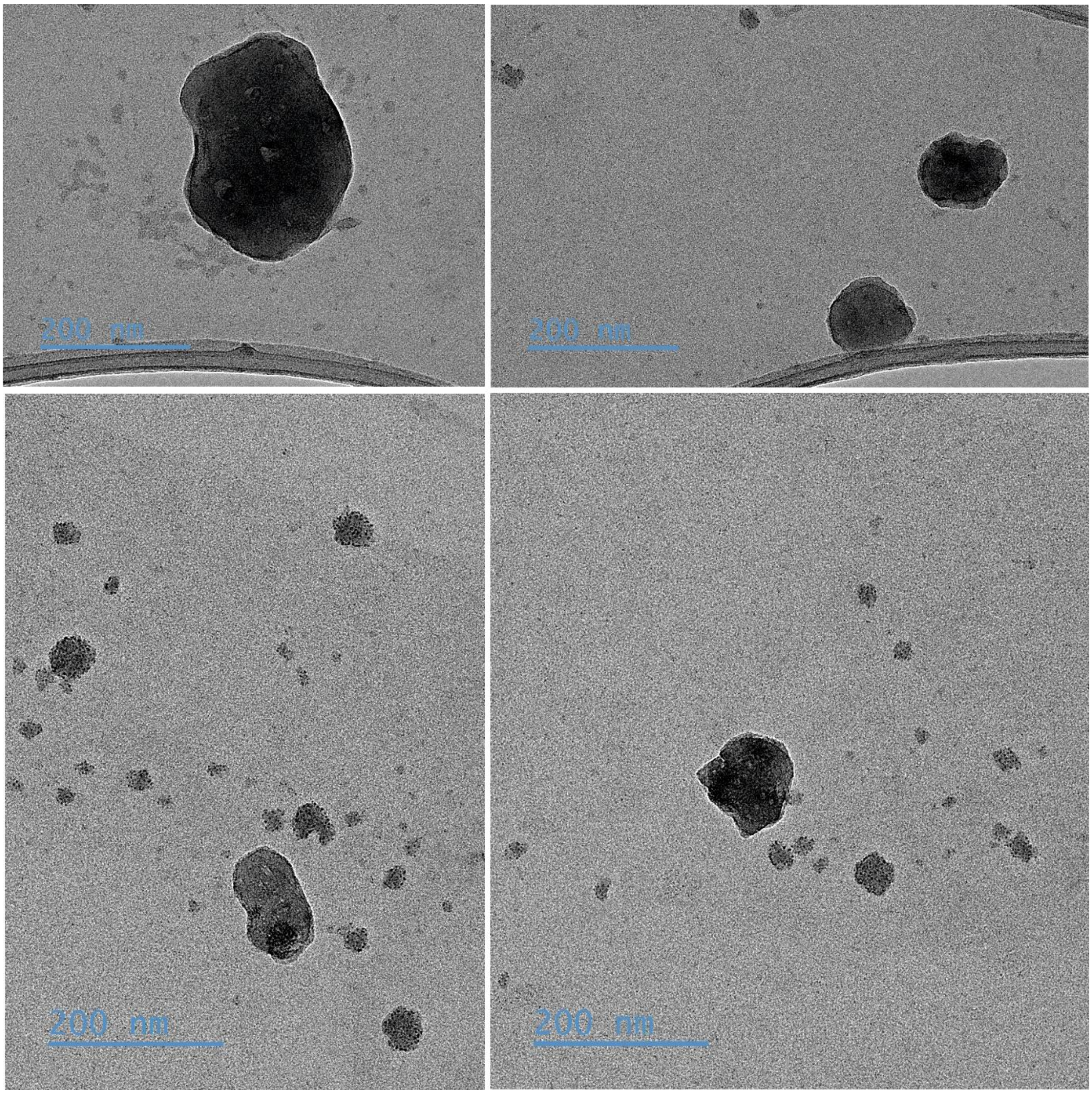

Fig. S2. Wide-field transmission electron microscopy (TEM) image of **$ZnSO_2$tBu-P123** (scale bar: 200 nm). The micrograph shows spherical micellar nanostructures together with occasional larger loose agglomerates, consistent with the secondary fraction observed in the DLS intensity profile.

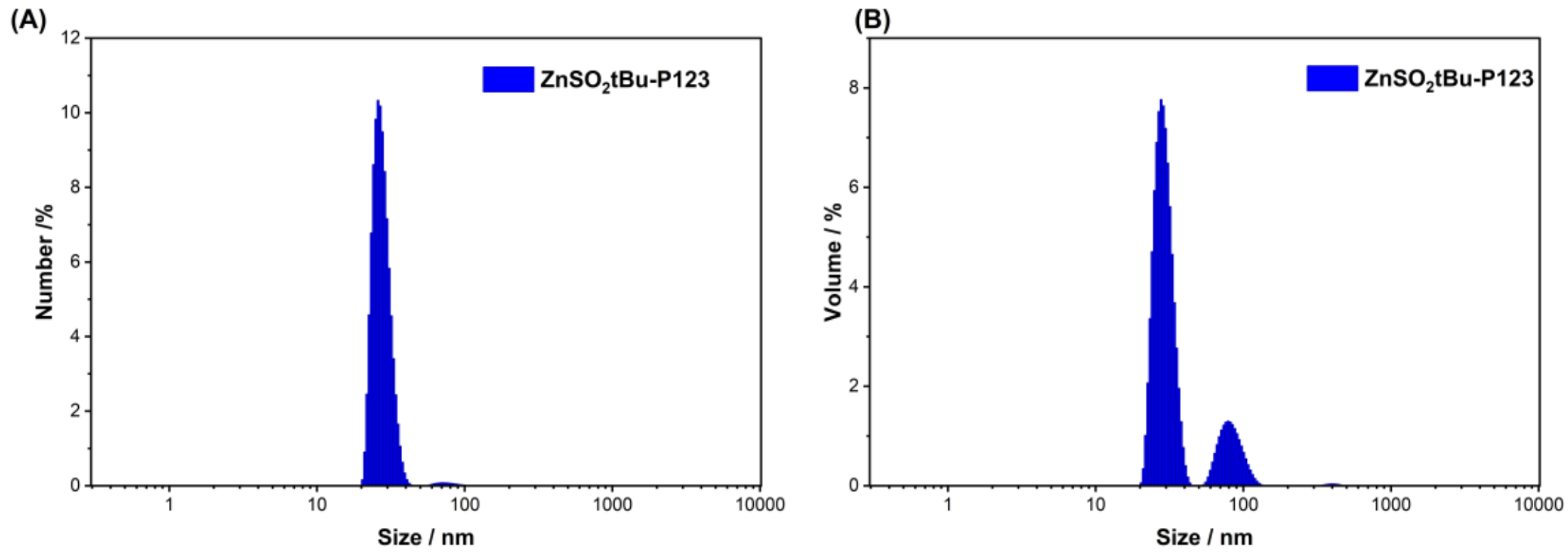

Fig. S3. Dynamic light scattering (DLS) size distribution profiles of the **$ZnSO_2tBu$-P123** micellar formulation in PBS. (A) Number-weighted distribution and (B) volume-weighted distribution. The number distribution is dominated by the primary nanoscale micellar population, while the volume distribution resolves a minor larger fraction, consistent with the secondary population observed in the DLS intensity profile.

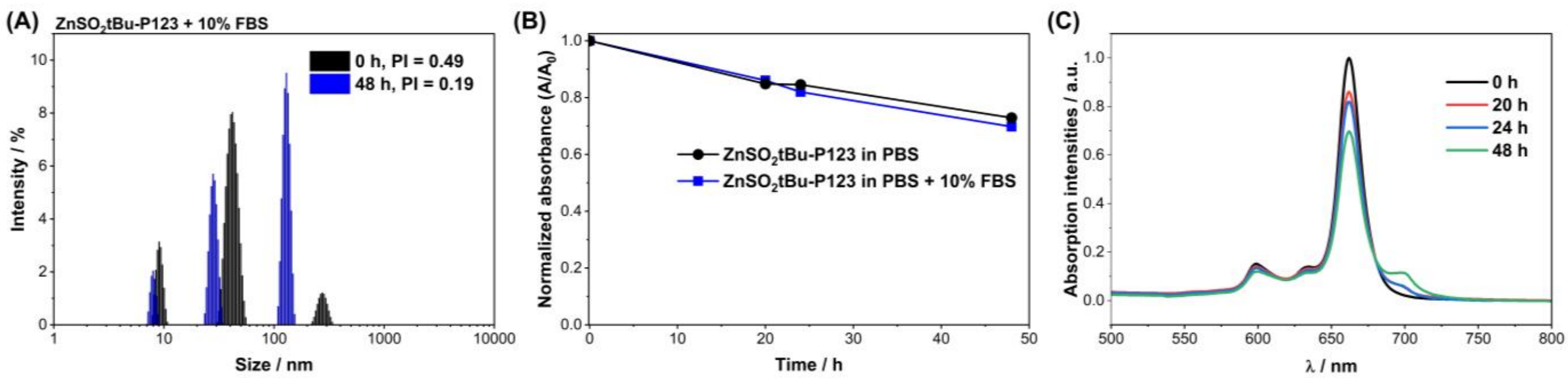

Fig. S4. Time-dependent colloidal and optical stability of the **$ZnSO_2tBu$-P123** formulation incubated at room temperature in the dark. (A) Intensity-weighted DLS profiles of **$ZnSO_2tBu$-P123** micelles dispersed in PBS supplemented with 10% fetal bovine serum (FBS) at 0 and 48 h, with corresponding PDI values. (B) Normalized Q-band absorbance of **$ZnSO_2tBu$-P123** in PBS and 10% FBS over time. (C) Electronic absorption spectra of **$ZnSO_2tBu$-P123** in 10% FBS at indicated time points, showing moderate hypochromicity with preservation of Q-band shape and no severe broadening or spectral shifts.

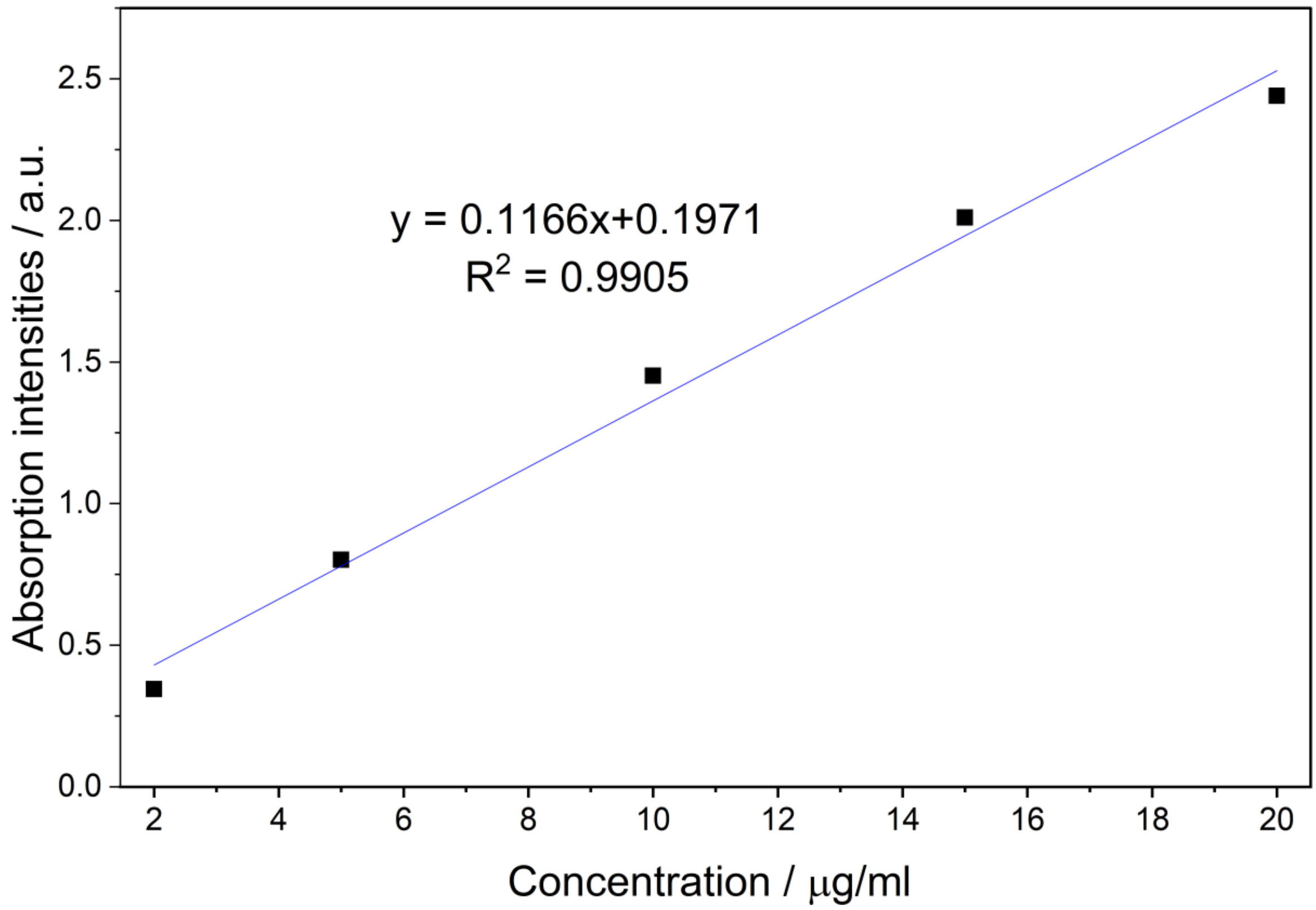

Fig. S5. Calibration curve for the determination of Encapsulation Efficiency (EE) based on the UV-Vis absorbance of **$ZnSO_2tBu$** in DMSO.

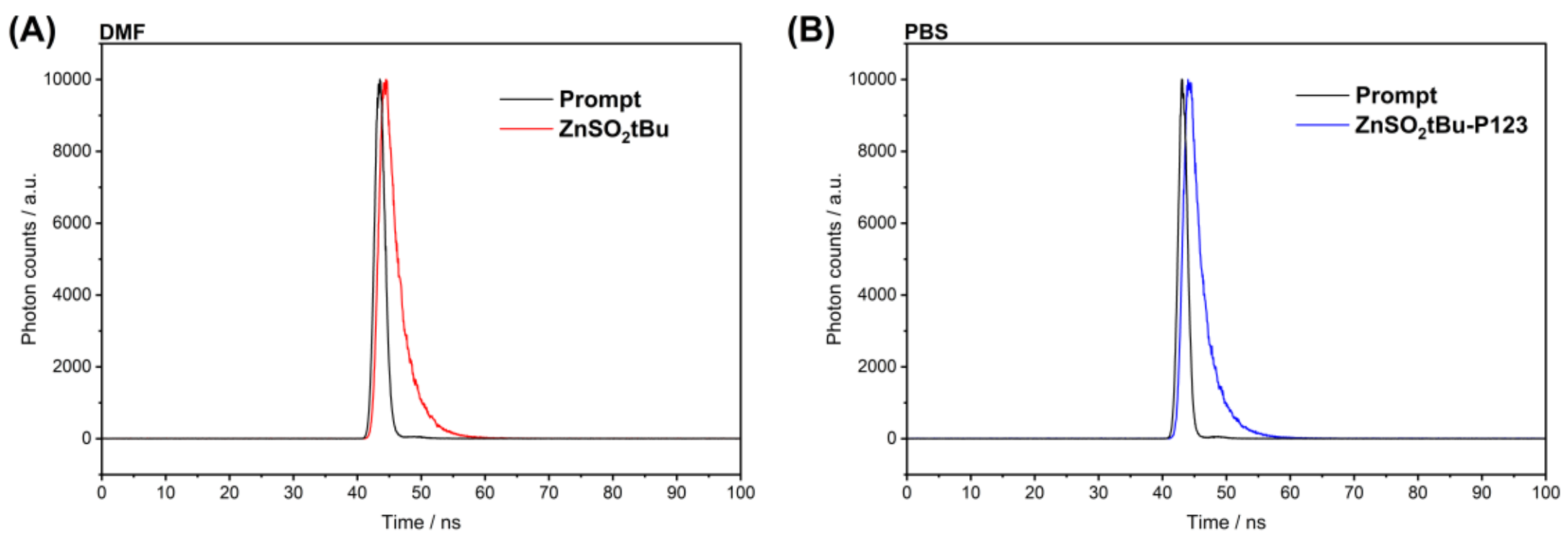

Fig S6. Time-resolved fluorescence decay profile for (A) **$ZnSO_2tBu$** registered in DMF and (B) **$ZnSO_2tBu$-P123** registered in PBS at room temperature.

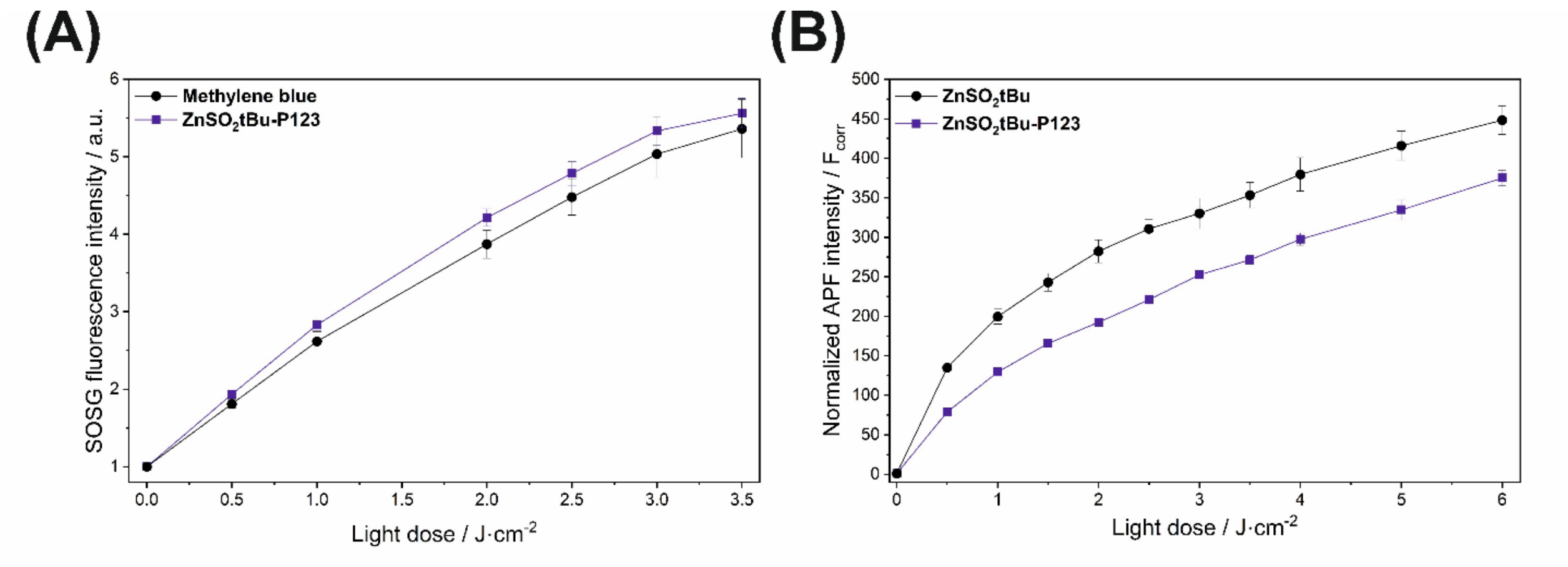

Fig. S7. (A) Singlet oxygen generation monitored by the increase in Singlet Oxygen Sensor Green (SOSG) fluorescence at 525 nm. The plot shows the linear correlation of fluorescence intensity versus irradiation time for **$ZnSO_2tBu$-P123** and the reference photosensitizer (Methylene blue). (B) APF oxidation as a probe-based readout of highly reactive ROS associated with Type I photochemistry. To account for the differences in light absorption between the formulations, the relative APF fluorescence increment (F/F_0 - 1) was normalized to the fraction of light absorbed (1 - 10^{-A}) by each sample at the irradiation wavelength. Both samples contained equimolar dye concentrations (17.6 µg/mL).

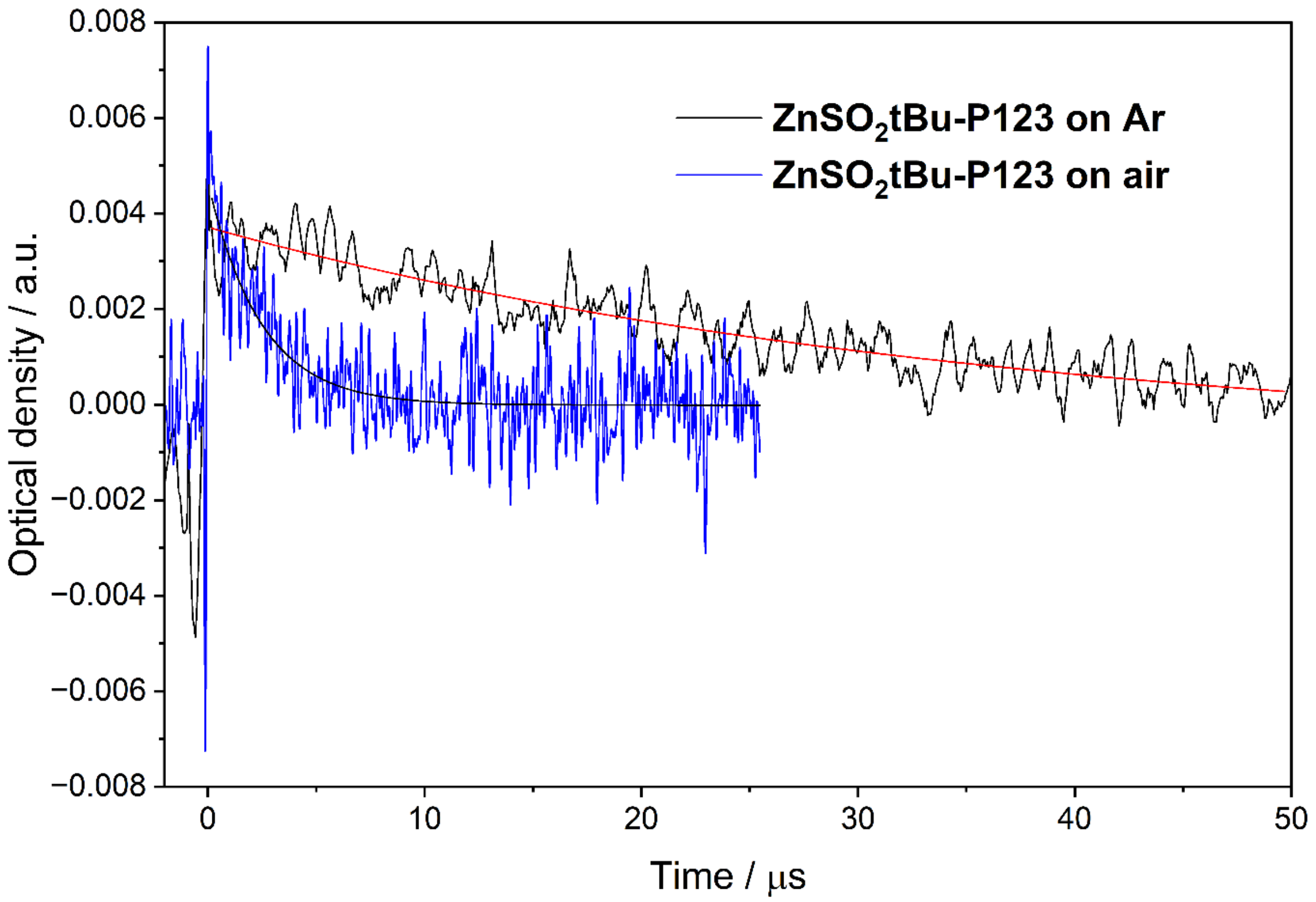

Fig. S8. Triplet-state decay profiles of the **$ZnSO_2tBu$-P123** micellar formulation under air- and argon-saturated conditions. Solid lines represent the monoexponential fits applied to the baseline-corrected experimental data.

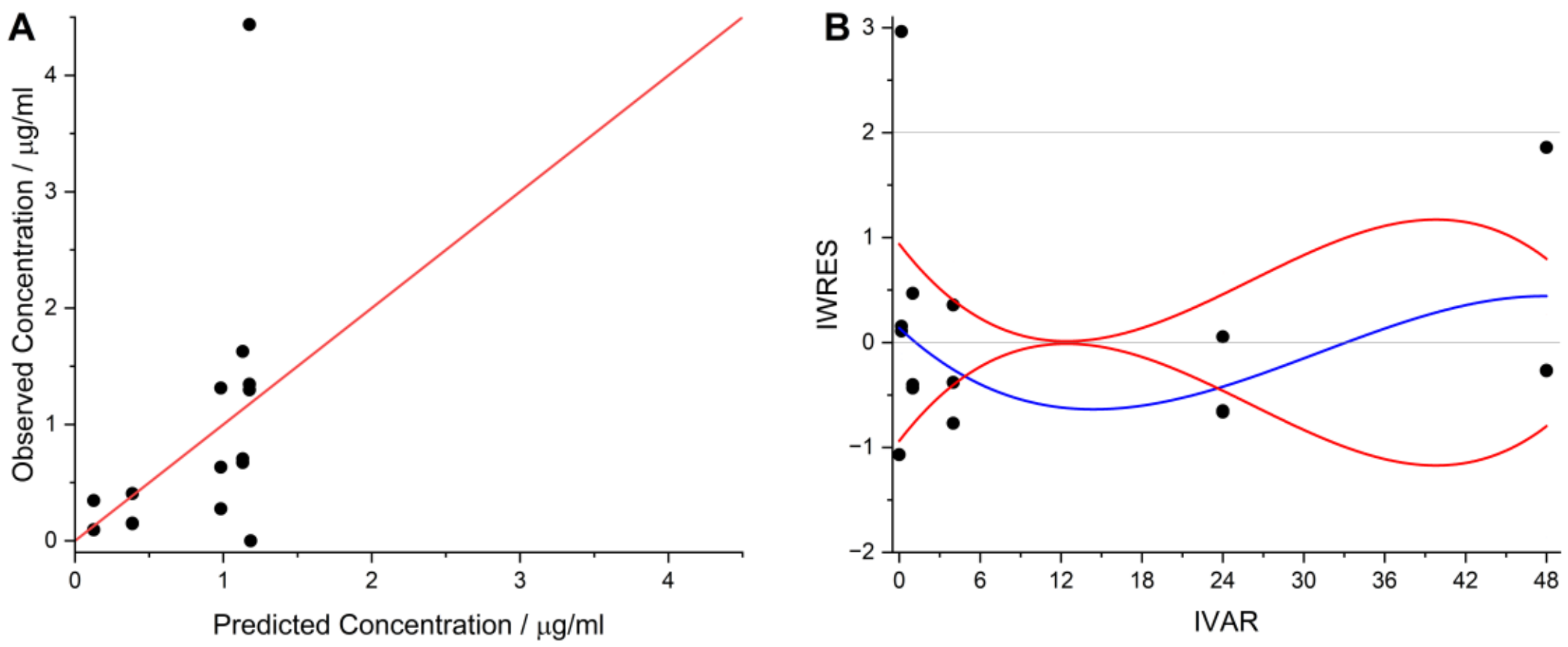

Fig. S9. Goodness-of-fit diagnostic plots for the pharmacokinetic modeling of **ZnSO$_2$tBu-P123** in mouse serum analyzed via Phoenix WinNonlin. (A) Observed versus predicted photosensitizer concentrations. (B) Individual weighted residuals (IWRES) versus independent variable (IVAR, time).

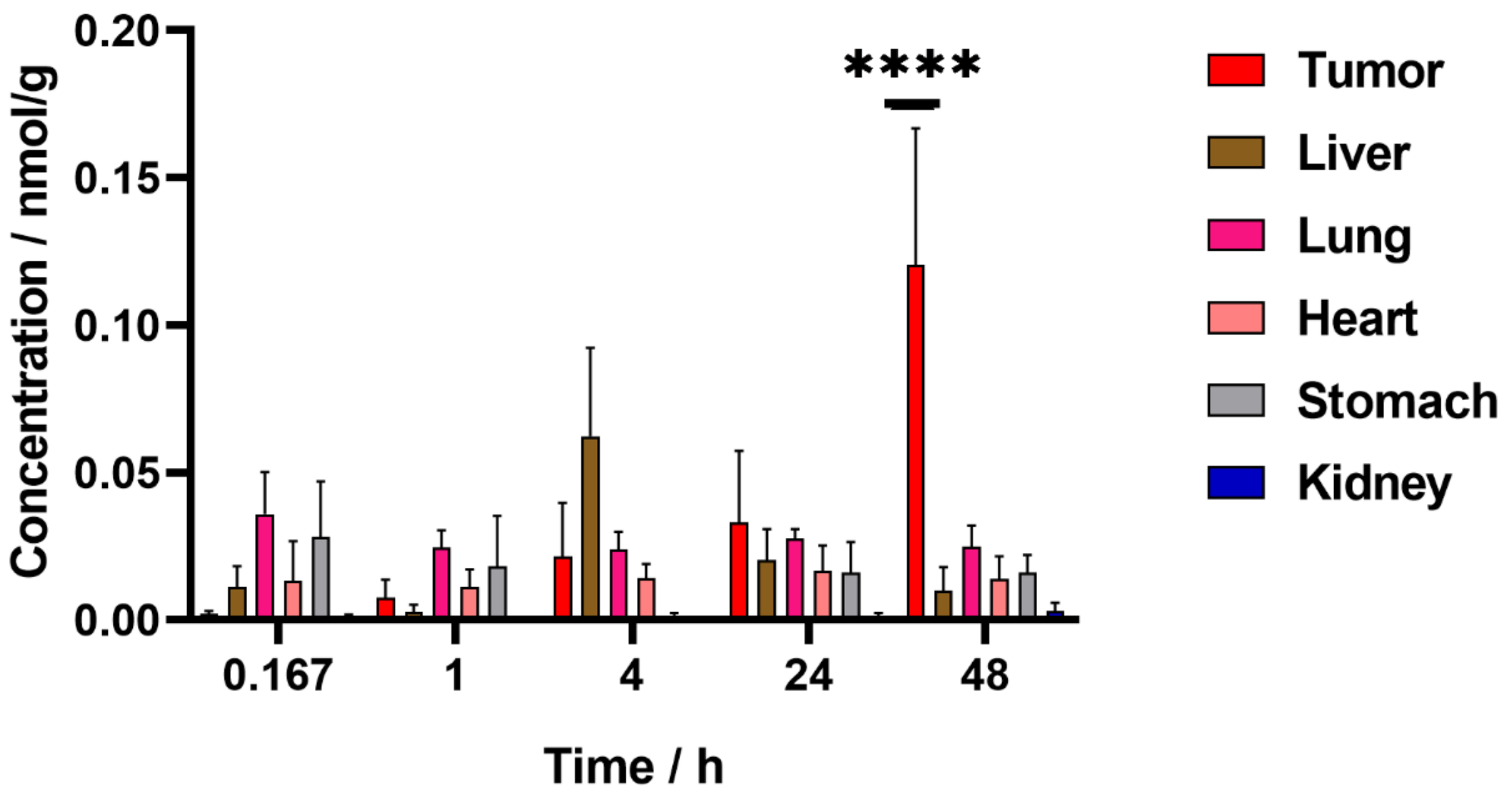

Fig. S10. Time-dependent *ex vivo* biodistribution of **ZnSO$_2$tBu-P123** in mice bearing CT26 tumors (mean ± SEM, n=3). Two-way ANOVA with Dunnett's multiple comparisons test comparing tumor uptake against other organs at each time point. At 48 h, tumor accumulation was greater than in all other analyzed organs (****$p < 0.0001$)

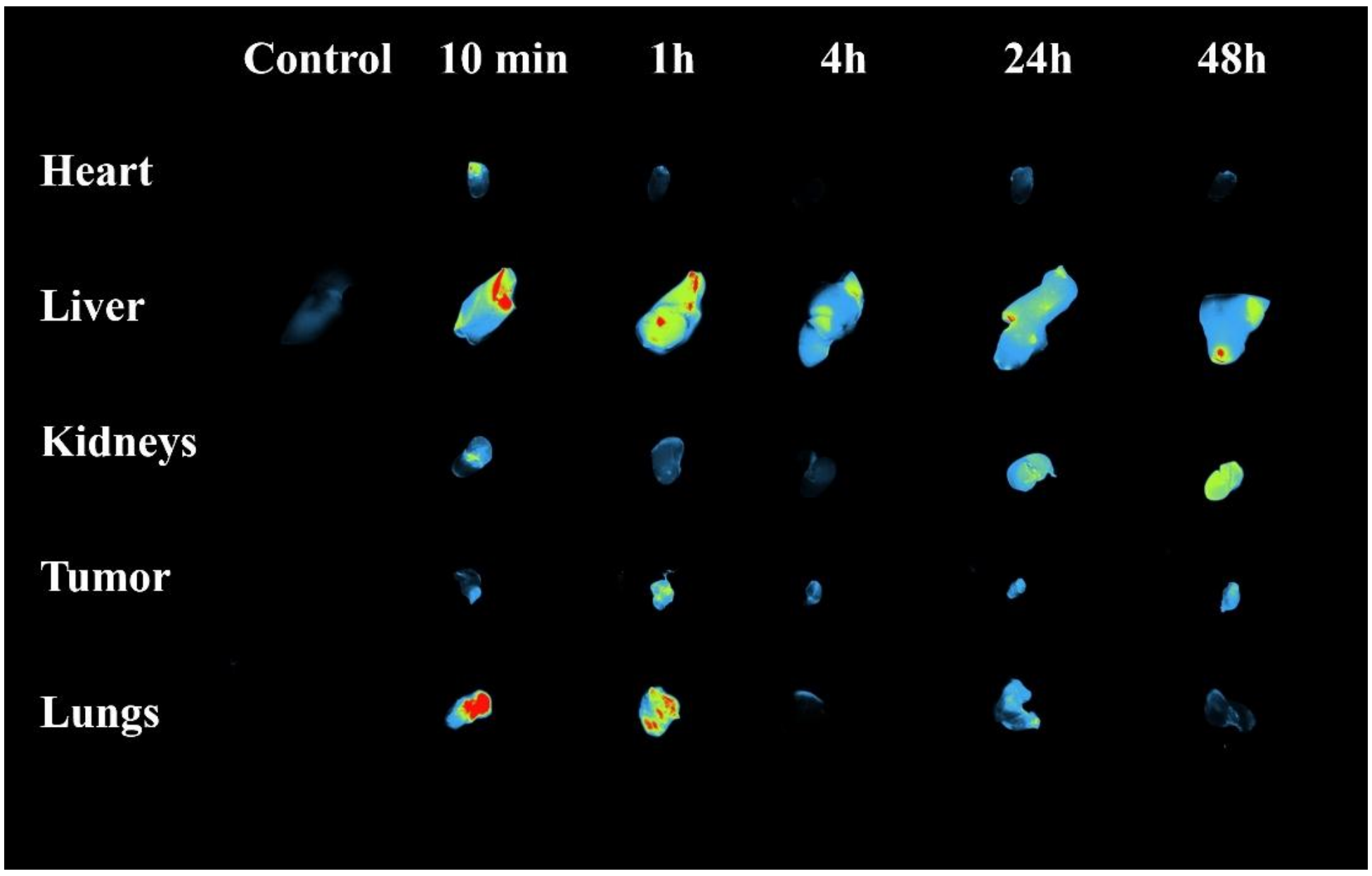

Fig. S11. *Ex vivo* fluorescence imaging of harvested organs (heart, liver, kidneys, tumor, lungs) isolated from BALB/c mice bearing CT26 tumors at designated time points (control, 10 min, 1 h, 4 h, 24 h, 48 h) following intravenous injection of **$ZnSO_2$tBu-P123**.

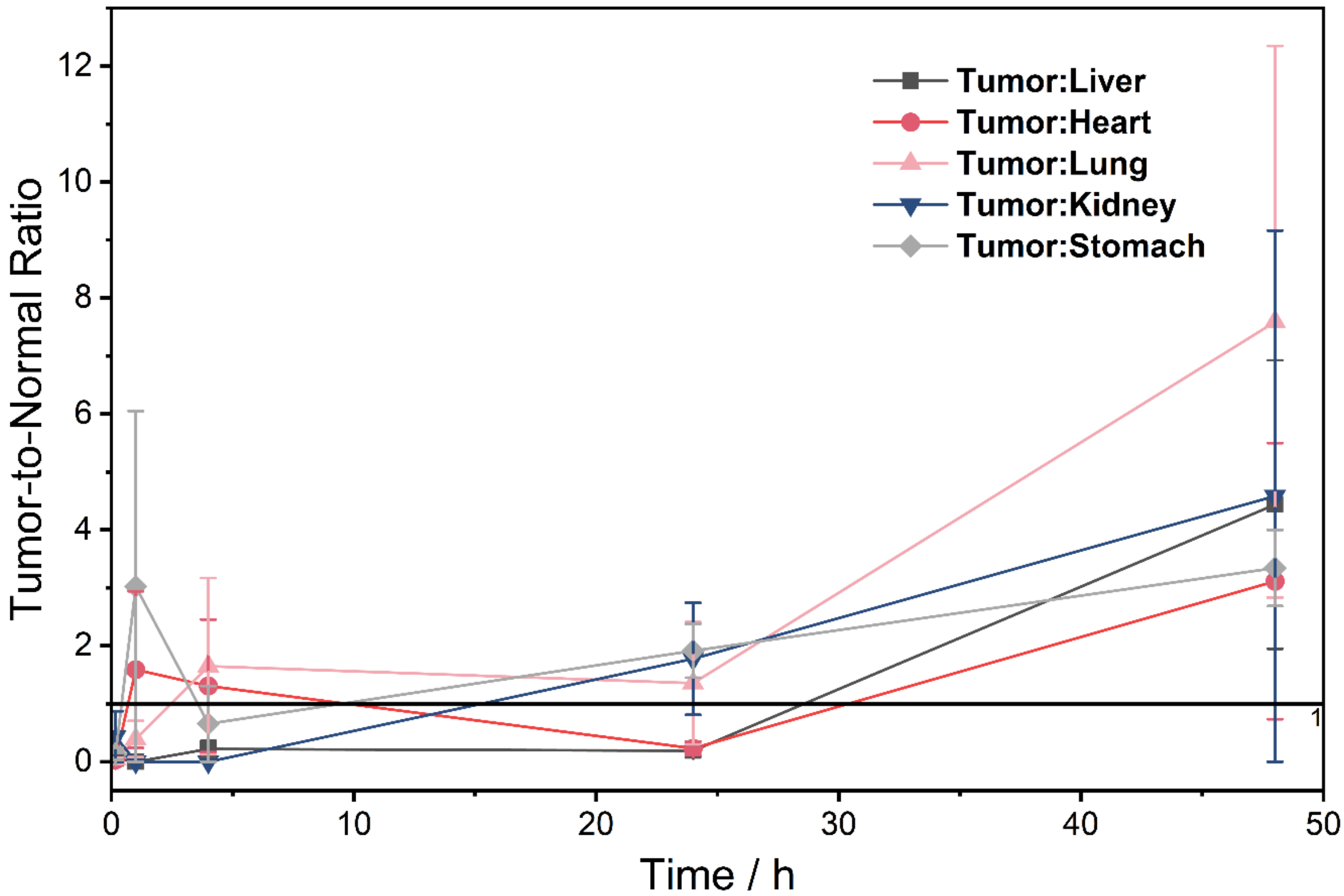

Fig. S12. Tumor-to-normal tissue concentration ratios (TNR) over time for liver, heart, lung, kidney, and stomach, calculated from the quantitative tissue distribution data. The profiles show progressive improvement in tumor selectivity, with favorable tumor-to-organ ratios already present at 24 h and further increases observed at 48 h post-injection.
